# Self-management of heart failure in dementia and cognitive impairment: a systematic review

**DOI:** 10.1186/s12872-019-1077-4

**Published:** 2019-04-29

**Authors:** Janaka Lovell, Tony Pham, Samer Q. Noaman, Marie-Claire Davis, Marilyn Johnson, Joseph E. Ibrahim

**Affiliations:** 1 Department of Forensic Medicine, Monash University, 65 Kavanagh Street, Southbank, Victoria, 3006 Australia; 2 Department of Cardiology, Alfred Health, Victoria, 3004 Australia; 3 Calvary Health Care Bethlehem, Victoria, 3162 Australia; 4 Institute of Transport Studies, Monash University, Victoria, 3800 Australia

**Keywords:** Aging, Self care, Heart failure, Dementia, Cognitive impairment, Cognitive domains

## Abstract

**Background:**

The cornerstone of effective management in heart failure (HF) is the ability to self-care.

Aims include i) To determine factors influencing self-care in HF patients with cognitive impairment (CI) and ii) to determine the influence of cognitive domains on self-care in patients with HF and CI.

**Methods:**

MEDLINE, CINAHL, EMBASE, EBSCOHost, PsychINFO, ProQuest Research Library, Health Technology Assessment Database, The Cochrane Library, Web of Science and Scopus databases were systematically searched. Original research describing the relationship between cognition and HF self-care in community-dwelling older persons with dementia/CI in English, published in a peer-reviewed journal from 1^st^January(2000)-22^nd^March(2016) was identified. Study and population characteristics, data sources, self-care processes, methods of cognitive assessment, cognitive domains affected, study outcomes, impact of impairment, and other risk factors of self-care impairment were abstracted by two reviewers.

**Results:**

Of 10,688 studies identified, 14 met the inclusion criteria. Patients with HF and CI ranged from 14 to 73%. Where reported, self-care maintenance adequacy ranged from 50 to 61%; self-care management adequacy ranged from 14 to 36% and self-care confidence adequacy ranged from 0 to 44% on the Self-care of Heart Failure Index (SCHFI). All but one study predicted poor self-care ability according to poor outcome on cognitive testing. Additionally, specific cognitive domain deficits impaired self-care. Subjects with lower cognitive scores were less likely to seek assistance while subjects with depression had poor self-care abilities.

**Conclusions:**

Clinicians must consider the type and severity of impairments in cognitive domains to tailor management. Awareness of depression, self-confidence and support access may modulate self-care ability.

**Electronic supplementary material:**

The online version of this article (10.1186/s12872-019-1077-4) contains supplementary material, which is available to authorized users.

## Background

Heart failure (HF) is a complex clinical syndrome arising from limited cardiac filling or ejection [1]. HF is a major economic burden estimated to cost the United States healthcare system approximately $USD 30.7 billion annually [1, 2]. HF has a prevalence of 1–2% in the general population however, rises to ≥10% of those above 70 years of age and contributed to one-in-nine deaths in 2009 [3]. With an ageing population, the prevalence of HF is projected to rise, which requires considering the management of patients with HF in the context of other comorbid geriatric conditions such as dementia [4].

Dementia (a severe form of cognitive impairment) is expected to double in prevalence every 20 years, reaching an estimated 74.7 million persons worldwide by 2030 [5]. Cognitive impairment (CI) is already commonplace being present in 25–75% of those with HF [6].

Patient self-care is a cornerstone of effective HF management. Key self-care behaviors in HF comprise adhering to complex medication regimens, ensuring dietary sodium and fluid restrictions, appropriate exercise as well as recognizing, managing, and seeking health care advice when changes in symptoms arise [7, 8].

Self-care in HF is a cognitively demanding process requiring response to cues, decision making, disease knowledge and skills in self-management tasks [6]. The neuropsychological deficits of attention, memory and executive dysfunction observed in HF patients could be related to neuroanatomical regional blood flow reduction and may challenge engagement in appropriate self-care behavior [9, 10]. Unfortunately, CI and ability to self-care is frequently overlooked, whereby sub-optimal engagement in HF self-care is assumed to be due to poor motivation and/or poor compliance [6, 8].

At present there are several proposed self-management programs for patients with HF, however, none benefit morbidity or mortality [11]. The impact of CI or dementia on participation is unknown due to paucity of data. A previous systematic review identified a significant correlation between mild cognitive impairment (MCI) and self-care in HF among eight of nine studies [6]. However, this only included populations with MCI. The current study is the first to examine self-care in community dwelling older persons with mild to severe CI.

### Aim

This systematic review aims to determine: i) factors influencing self-care in HF patients with dementia/CI ii) whether deficits in specific cognitive domains have a differential influence on HF self-care in patients with a co-morbid dementia/CI.

## Methods

This review was conducted according to the Preferred Reporting Items for Systematic Reviews and Meta-Analyses (PRISMA) (Additional file 1) [12].

### Definitions

In this review, dementia is defined as a significant cognitive decline from baseline performance in one or more of five cognitive domains: complex attention, language, perceptual-motor function, learning and memory and, executive function (Additional file 1: Table S1) with concomitant impairment in independent functioning. MCI is defined as a non-normal, non-demented cognitive state with decline from baseline performance in one or more of six cognitive domains, where the deficits do not impair independent functioning [13]. Cognitive impairment in this review is defined as a clinical state encompassing any degree of CI from mild to severe (dementia).

Self-management is defined as the ability of the patient to be an active participant in their treatment where they arereponsible for daily management. Self-management comprises five core skills: problem solving, decision making, resource utilization, interacting with health care providers and, taking action [14]. We also describe domains of self-care identified in the Self Care of Heart Failure Index (SCHFI) including self-care maintenance (10 items: behavioural adherence to treatment recommendations), self-care management (6 items: ability to recognise symptoms and respond appropriately by implementing remedies and determining their effectiveness) and self-care confidence (6 items: confidence to engage with self-care processes) [15].

The definition of HF is from the American Heart Association/American College of Cardiology as a *“complex clinical syndrome that can result from any structural or functional cardiac disorder that impairs the ability of the ventricle to fill or eject blood”* [1].

### Data sources and searches

The following ten databases were searched on 22 March 2016: MEDLINE, CINAHL, EMBASE, EBSCOHost, PsychINFO, ProQuest Research Library, Health Technology Assessment Database, The Cochrane Library, Web of Science and Scopus.

Key terms describing dementia, an aged population, HF and self-management were identified by JEI and JL (Additional file 1), adapted to each database and used to conduct a systematic search. A bibliographic review of included articles was conducted identify additional relevant studies.

### Study selection

Inclusion criteria comprised original research available in English published in a peer-reviewed journal from 1 January 2000 to 22 March 2016. The study population of interest was community dwelling older persons. Included studies investigated paradigms of self-management in populations who had an established diagnosis of dementia or MCI. Studies exploring the impact of dementia or MCI, and the influence of differentially impaired cognitive domains on self-management in HF persons were included.

Exclusion criteria comprised studies that included populations without CI or populations without a diagnosis of HF. Studies solely testing the effect of an intervention could have introduced bias and therefore were excluded.

MJ and JL screened results for eligibility by title and abstract. TP and JL then independently applied inclusion and exclusion criteria to the full texts to select studies to be appraised, and final selection was made by consensus between JL, TP and JEI.

### Data extraction and quality assessment

Extracted information consisted of study and population characteristics, data sources, self-care processes, methods of cognitive assessment, cognitive domains affected, study outcomes, impact of impairment, and other risk factors of self-care impairment. Conversion of scales reporting the severity of comorbid conditions was developed.

Included articles were independently assessed by TP and JL using the National Institutes of Health (NIH) study quality assessment tool and differences were resolved by discussion.

### Role of the funding source

This work was supported by the Australian Government Dementia Training Study Centers, Monash University and Ballarat Health Services. These organizations did not have a role in study selection, quality assessment, data synthesis, or in the writing of the manuscript. The investigators are solely responsible for the content of the review.

## Results

### Study and population characteristics

The combined searches returned 10,688 studies, of which 14 met the inclusion criteria (Additional file 1). Of the 14 studies, just over a third (*n* = 6) of the studies were based in the United States of America (USA), two studies were conducted in each of Sweden and Australia while one study was conducted in each of Korea, Italy, Canada and the Netherlands. All studies (*n* = 14) were published from 2005 onwards (Table 1). Included studies were judged to be of fair (*n* = 9) and good (*n* = 5) quality.

**Table 1:** Study and Population Characteristics

Author	Country	Aim	Study Duration (months)	Data Type	Study Design	Method(s) of data collection	Setting	Population setting	Population size (n)	Age mean and range	Female (n and/or %)	HF severity (n and/or %)	Cognitive impairment test(s) and cutoff scores	Cognitive Impairment (n and/or %)	Comorbidities (n and/or %)*	Quality Assessment
Alosco, 2012	USA	To examine whether cognitive functioning is associated with poorer Adh to treatment recommendations	–	Cross Sectional	Obs	Ques, Exam	Primary Care/Cardiology Practice	Urban	149	68.1 (SD = 10.7)	37%	NYHA II/III LVEF: 41.0 (SD = 14.8)	–	–	Diabetes: 34% Depression: 22% Hypertension: 72% Myocardial Infarct: 52%	Fair
Alosco, 2012	USA	To examine whether cognitive functioning is able to predict ADL performance	–	Cross Sectional	Obs	Ques, Exam	Primary Care/Cardiology Practice	Urban	122	68.5 (SD = 9.4)	35%	NYHA II/III	MMSE	–	Diabetes: 33% Hypertension: 66% Myocardial Infarct: 54%	Fair
Alosco, 2014	USA	To examine the association between EF and IADL in HF patients & to examine the association between executive dysfunction and unhealthy lifestyle behaviors.	–	Cross Sectional	Obs	Ques, Exam	–	Urban	179	68.1 (SD = 10.3)	36%	NYHA II/III/IV LVEF: 41.0 (SD = 15.1)	–	–	Diabetes: 37% Hypertension: 70%	Fair
Cameron, 2009	AUS	To test a conceptual model of factors drawn from the literature as determinants of chronic HF SC	–	Cross Sectional	Obs	Int	Inpatient	Urban	50	73 (SD = 11)	12 (24%)	NYHA III/IV: 25 (50%)	MMSE (< 27)	18 (36%)	Mild/Moderate: 32 (64%) Severe: 18 (36%)	Good
Dickson, 2008	USA	To explore how attitudes, self-efficacy and cognition influence the decision making processes underlying HF SC.	–	Cross Sectional	Obs	Int	Outpatient	Urban	41	49.2 (SD = 10.5) Range: 25–65	15 (37%)	NYHA II/III Mean ejection fraction: 34%	–	–	Mild: 17 (41%) Moderate: 20 (49%) Severe: 4 (10%)	Fair
Habota, 2015	AUS	To compare prospective memory ability of CHF patients and matched controls	3	Cross Sectional	Obs	Int	Outpatient	Urban	30	70.0 (SD = 11.9) Range: 40–86	37%	NYHA III/IV: (30%)	ACE-R	–	Diabetes: 5 (17%) Hypertension: 20 (67%)	Fair
Harkness, 2014	CAN	To determine if MCI was significantly associated with SC management in a community dwelling sample of older HF patients	–	Cross Sectional	Obs	Ques, Exam	Outpatient	Urban	100	72.4 (SD = 9.8)	32%	NYHA III: 43 (43%) LVEF≤45: 90%	MoCA (< 26, < 24 – CVS cutoff)	< 26: 73% < 24: 56%	AF: 54 (54%) Diabetes: 43 (43%) Depression: 12 (12%) Hypertension: 73 (73%)	Good
Hawkins, 2012	USA	To describe the prevalence and severity of CI in an OP veteran population with HF and to describe the cognitive domains affected. To examine the clinical and demographic variables associated with CI and to determine the relationship between CI and MA	–	Prospective	Coh	Int, Exam	Outpatient/General Medical Clinic	Urban	251	66 (SD = 9.8) Range: 33–93	4 (1.6%)	LVEF: 37.5 (SD = 16.9)	SLUMS (< 27 with HSQ, < 25 with-out)	144 (58%)‡	AF: 82 (32.7%) Diabetes: 134 (53.4%) Depression: 76 (30.3%)‡ Hypertension: 193 (76.9%)	Good
Hjelm, 2015	SWE	To a) test the association between cognitive function and SC in HF patients, b) explore which cognitive areas were affected, c) determine if DP moderated the association between cognitive function and SC.	–	Cross Sectional	Obs	Ques, Exam	Outpatient	Urban	142	Median: 72, Range: 65–79	45 (32%)	NYHA III/IV: 55 (39%) LVEF< 40: 102 (72%)	MMSE	–	Mild: 116 (82%) Moderate: 22 (15%) Severe: 3 (2%)	Good
Karlsson, 2005	SWE	To assess the effect of a nurse based management program to increase HF patients’ knowledge about disease and SC. To compare these results to gender and cognitive function	6	Prospective	RCT	Ques, Int	Outpatient	Urban	Interv: 72 Control: 74	76, SD = 8 vs. 76 SD = 7§	31 (43%) vs. 33 (45%)§	NYHA III/IV: 31 (43%) vs. 22 (30%)§ LVEF: 33 (SD = 12) vs. 35 (SD = 10)§	MMSE	–	Diabetes: 17 (24%) vs. 15 (20%)§ Hypertension: 30 (42%) vs. 21 (28%)§||	Fair
Kim, 2015	KOR	To examine a) global cognition, M and EF, b) differences in these domains when comparing asymptomatic and symptomatic HF c) the association between cognitive function and SC Adh in HF patients d) the influence of the cognitive domains on MACE	24	Prospective	Coh	Int	Outpatient	Urban	86	58.3 (SD = 12.9)	28 (34%)	NYHA III/IV: 8 (9%) LVEF: 51 (SD = 15)	K-MMSE (< 23.5)	28 (33%)	AF: 15 (17%) Diabetes: 13 (15%)	Fair
Lee, 2013	USA	To quantify the relationship between MCI and, SC and consulting behaviours	–	Cross Sectional	Obs	Ques, Exam	Outpatient	Urban	148	56.9 (SD = 12.4)	57 (39%)	NYHA III/IV: 87 (59%) LVEF: 28 (SD = 12)	MoCA (< 26, < 24 – CVS cutoff)	< 26: 49 (33%) < 24: 21 (14%)	Mild: 95 (64%) Moderate: 44 (30%) Severe: 9 (6%)	Good
Smeulders, 2010	NED	To identify the characteristics of CHF patients that benefitted most from the CDSMP	27	Prospective	RCT	Ques, Int (T)	Outpatient	Urban	Interv: 186 Control: 131	66.7 (SD = 10.6), 66.6 (SD = 11.0) vs. 66.8 (SD = 10.1)§	45 (24.2%) vs. 42 (32.1%)§	NYHA III: 66 (36%) vs. 40 (31%)§	TICS (< 33.0)	99 (53.2%) vs. 78 (59.5%)§	–	Fair
Vellone, 2015	ITA	To determine whether SC confidence mediates the relationship between cognition and SC behaviours	–	Cross Sectional	Obs	Int	Outpatient	Urban	628	73.0 (SD = 11.3)	266 (42.6%)	NYHA III/IV: 340 (54.1%) LVEF: 43.1 (SD = 11.6)	MMSE	–	–	Fair

* Classified as mild, moderate, severe as in Additional file 1. If the measures were not available, prevalence of atrial fibrillation, diabetes, depression, hypertension and myocardial infarction were reported where available

‡ Denominator is 250

§ Intervention vs. Control

|| *p* < 0.05

**Country:** AUS = Australia, CAN=Canada, ITA = Italy, KOR = South Korea, NED = Netherlands, SWE = Sweden, USA = United States of America

**Study design:** Obs = Observational, Coh = Cohort, RCT = Randomized Controlled Trial

**Method of data collection:** Exam = Examination, Int = Interview (T = Telephone), Ques = Questionnaire,

**Population size:** Interv = Intervention

**Heart failure severity:** LVEF = Left ventricular ejection fraction, NYHA = New York Heart Association,

**Cognitive tests:** 5WIDM = 5 Word Immediate and Delated Memory Test, ACE-R = Addenbrooke’s Cognitive Examination, CVS=Cardiovascular, HSQ = High school qualification, K-MMSE = Korean Mini Mental State Exam, MMSE = Mini Mental State Exam, MoCA = Montreal Cognitive Assessment, SLUMS=St Louis University Mental Status, TICS = Telephone Interview for Cognitive Status

**Comorbidities:** AF = Atrial fibrillation

**Other:** Adh = self-reported adherence, ADL = Activities of daily living, CDSMP=Chronic Disease Self-Management Programme, CHF=Congestive heart failure, CI=Cognitive impairment, DP = Depression, EF = Executive function, HF=Heart failure, IADL = Instrumental activities of daily living, MA = Medication adherence, MCI = Mild cognitive impairment, MACE = Major adverse cardiac events, OP=Outpatient, SC=Self-care

Included studies utilized various measures to ascertain HF self-care including disease knowledge (*n* = 1) (16], The Kansas City Cardiomyopathy Questionnaire (KCCQ) (n = 1) (17], prospective memory (*n* = 1) [18], adherence to prescribed medication and lifestyle regimens (*n* = 2) [19, 20], ability to complete activities of daily living (ADLs) and independent activities of daily living (IADLs) (*n* = 2) [21, 22], The European Heart Failure Self-Care Behavior Scale (EHFScB-9) (n = 1) [23, 24] and The SCHFI (*n* = 6) [9, 25–32] of which one [31] study also utilized the EHFScB-9 (Table 1).

Study designs were largely cross sectional (*n* = 10). Other designs included prospective cohort studies (*n* = 2) and randomized controlled trials (n = 2). Methods of data collection included questionnaires in combination with examination (*n* = 6), face-to-face interview (n = 1) or telephone interview (n = 1), face-to-face interview alone (*n* = 5) or interview in combination with examination (*n* = 1). Overall study populations were large, ranging from 30 [33] to 628 [32] participants. Participants were mostly hospital outpatient attendees (*n* = 10) while the remaining participants were from primary care/cardiology clinics (*n* = 2), a mix of hospital inpatients and external sources (*n* = 1) while one study [22] did not identify the setting from which participants were drawn (Table 1).

Most studies had participants with a mean age over 65 years (*n* = 11). Studies comprised predominantly male participants with proportions ranging from 55% [16] to 98.4% [20] (Table 1). All studies utilized populations from an urban setting.

The severity of HF for included patients were reported according to the New York Heart Association (NYHA) classification for HF and/or left ventricular ejection fraction (LVEF). Three studies [19, 21, 22] recruited subjects with NYHA class II to IV. Patients in these studies had a mean LVEF of 41%. One study recruited subjects with NYHA classes II and III whereby patients had a mean ejection fraction of 34% [9]. In studies with HF patients as a subgroup, HF made up 9–59% of subjects and patients either had NYHA class III or IV [16, 17, 24, 25, 29–32]. In many studies, when reported, measured LVEF ranged from 28 to 51% [16, 20, 29–32] with one study reporting 90% of subjects having a LVEF ≤45% [29] and another with 72% of the study population having a LVEF < 40% [24].

Twelve studies reported the comorbidity status of their subjects. Where comorbidity scores were convertible to severities (Additional file 1), the reported severity of comorbidities ranged from mild to severe [9, 24, 25, 31]. Mild comorbidity severity was prevalent in 41–82%, moderate severity in 15–30% and severe severity in 2–36% of these studies’ populations. Commonly reported comorbidities for HF patients in the community comprised hypertension (42% [16] - 76.9% [20]), previous myocardial infarction (52% [21] – 54% [19]), atrial fibrillation (17% [30] – 54% [29]) and diabetes (15% [30] – 53.4% [20]) (Table 1).

### Cognitive impairment

Assessment of cognition was achieved through a combination of examination (*n* = 7) or interview (*n* = 6), with one study utilizing a telephone interview (*n* = 1) [17] to assess neuropsychological status. Global cognition was assessed in most (*n* = 11) of the appraised studies. Tests used to measure global cognition varied and included Mini Mental State Examination (MMSE) (*n* = 5), Montreal Cognitive Assessment (MoCA) (*n* = 2), Korean version of the MMSE (K-MMSE) (*n* = 1), Addenbrooke’s Cognitive Examination (n = 1), St. Louis University Mental Status (SLUMS) Exam (*n* = 1), Probed Recall Memory Test (*n* = 1), Digit Symbol Substitution Test (DSST) (*n* = 1) and Telephone Interviews of Cognitive Testing (*n* = 1) (Table 2).

**Table 2:** Cognitive Domains and Self-Care Processes Affected in Study Populations

					Cognitive Domains					Self-care			
Author	Country	Method of neuropsychological testing	Assessment of Cognitive Impairment	Cognitive Impairment scores (mean)	Attention and Information Processing	Language	Visuospatial Ability and Praxis	Learning and Memory	Executive Function	Assessment of Self-care	Self-care maintenance	Self-care management	Self-care confidence
Alosco, 2012	USA	Exam	None	–	TMTA: 40.7 (SD = 14.9) DSC: 50.5 (SD = 14.2)	BNT: 53.5 (SD = 5.7) AFT: 19.5 (SD = 5.1)	TMTA: 40.7 (SD = 14.9)	CVLT: SDFR = 7.6, (SD = 3.2) LDFR: 8.1 (SD = 3.3) Recognition: 13.60 (SD = 2.05)	TMTB: 127.7 (SD = 77.2) LNS: 8.9 (SD = 2.5) SCWIE: 0.1 (SD = 7.4)	Treatment Adherence (Self-Reported)	Drs Appointment: (94.8/100, SD = 16.8): 3% Non-adherent^a^ Medication Management: (96.1/100, SD = 11.5) - 1% Non-adherent^a^ Diet: (69.8/100, SD = 24.0) - 32% Non-adherent^a^ Exercise: (57.7/100, SD = 33.1) - 49% Non- adherent^a^ Smoking Abstinence: (94.1/100, SD = 21.0) - 7% Non-adherent^a^ Alcohol Abstinence: (91.1/100, SD = 23.6) - 7% Non-adherent^a^	–	–
Alosco, 2012	USA	Exam	MMSE	27.7 (SD = 1.8)	TMTA: 39.0, (SD = 13.5)	–	TMTA: 39.0, (SD = 13.5)	–	TMTB: 115.8, (SD = 58.2)	Activities of Daily Living	Shopping (1.68/2.00, SD = 0.58) Food preparation (1.46/2.00, SD = 0.84) Feeding (1.98/2.00, SD = 0.13) Transport (1.94/2.00, SD = 0.23) Medication Management (1.91/2.00, SD = 0.34) Telephone Usage (1.98/2.00, SD = 0.20)	–	–
Alosco, 2014	USA	Exam	None	–	DSC: 49.2, (SD = 14.7)- 11% impaired^b^	AFT:19.1, SD = 4.9) – 3% impaired^b^	–	CFT: LDR 13.0, (SD = 6.2) - 9% impaired^b^	FAB: 15.5 (SD = 2.6) - 30% impaired^b^ LNS: 8.8 (SD = 2.5) - 6% impaired^b^	Instrumental Activities of Daily Living	Shopping - 27%^c^ Food Preparation - 32%^c^ Transport - 8%^c^ Medication Management - 6%^c^ Telephone Usage - 2%^c^	–	–
Cameron, 2009	AUS	Interview	MMSE	–	–	–	–	–	–	Self-Care Heart Failure Index	67.8/100 (SD = 17.3) 52% had adequate^d^ scores	50.1/100 (SD = 16.6), 12% had adequate^d^ scores	62.0/100 (SD = 20.0), 36% had adequate^d^ scores
Dickson, 2008	USA	Interview	None	–	DSS, LNS	–	DSS	DSS: PMR - 46.3% had impaired memory, LNS	LNS	Self-Care Heart Failure Index	71.6/100 (SD = 14.3), 61% had adequate^d^ scores	71.3/100 (SD = 18.6), 44% had adequate^d^ scores	–
Habota, 2015	AUS	Interview	ACE-R	90.8 (SD = 4.6)	–	–	–	WAIS-IV DS (working memory), RAVT (verbal memory) VW (prospective memory)	TMT (TMTB-TMTA) (cognitive flexibility) HSCT (inhibition) Verbal fluency from ACE-R (initiation)	Prospective Memory	Virtual Week (ability to recall daily tasks)	–	–
Harkness, 2014	CAN	Exam	MoCA	–	–	–	–	–	–	Self-Care Heart Failure Index	67.1/100 (SD = 16.0). 50% had adequate^d^ scores	51.1/100 (SD = 23.6), 21% had adequate^d^ scores	55.4/100 (SD = 20.0), 22% had adequate^d^ scores
Hawkins, 2012	USA	Exam	SLUMS	24.4 (SD = 4.0)	WAIS-IV DS: z = −0.60, SD = 0.88, (NS) and WAIS-IV LNS: z = −0.56, SD = 0.68, (NS) Trails A: z = − 0.80, SD = 0.99, (NS) RBANS coding: z = −1.20, SD = 0.87, (NS)	RBANS PN: z = 0.23, SD = 1.24, (NS) RBANS SF: z= − 0.86, SD = 0.88, (NS) AFT: z = − 0.57, SD = 1.17, (NS)	RBANS FC: z = 0.67, SD = 1.53, (NS) RBANS LO: z = 0.10, SD = 0.85, (NS) WAIS-IV MR: z = − 0.20, SD = 0.98, (NS)	RBANS LL: z = − 1.90, SD = 0.96, (S) RBANS SM: z = − 1.59, SD = 1.08, (S) RBANS LR: z = − 1.25, SD = 0.91, (NS) RBANS LRR: z = − 1.80, SD = 1.84, (S) RBANS SR: z = − 1.84, SD = 1.21, (S) RBANS RF: z = − 0.36, SD = 1.04, (NS)	COWA: z = − 0.74, SD = 0.90, (NS) Trails B: z = − 0.73, SD = 1.04, (NS) WAIS-IV similarities: z = − 0.17, SD = 0.70, (NS)	Medication Adherence	Medication Adherence: Normal vs. Mild cognitive impairment - 78.1% vs. 70.7%, *p* = 0.017, Mild cognitive impairment vs. dementia: 70.7% vs. 73.3%, *p* = 0.31	–	–
Hjelm, 2015	SWE	Exam	MMSE	–	TMTA	–	TMTA, ROCF, BDT	ROCF, MOS, WKT	TMTB	EHFScBS-9	EHFScBS-9 (under diet, medication adherence)	EHFScBS-9 (under symptom monitoring and recognition)	–
Karlsson, 2005	SWE	Interview	MMSE	Intervention vs. control: 26.8 (SD = 3.3) vs. 26.9 (SD = 3.0)	–	–	–	–	–	Heart Failure Knowledge	–	–	–
Kim, 2015	KOR	Interview	K-MMSE	26.4 (SD = 5.3)	–	–	–	Seoul VLT: IR:15.5 (SD = 5.8) - 65% < normal DR: 4.8 (SD = 2.3) - 65% < normal	COWA: 20.1 (SD = 10.2) - 61% < normal	Self-Care Heart Failure Index	55.4/100 (SD = 14.3) 15% had adequate^d^ scores	34.0/100 (SD = 12.8), 0% had adequate^d^ scores^e^	52.1/100 (SD = 17.6), 14% had adequate^d^ scores
Lee, 2013	USA	Exam	MoCA	–	–	–	–	–	–	Self-Care Heart Failure Index / EHFScBS-9	69.2/100 (SD = 14.3)	67.3/100 (SD = 19.0)	63.9/100 (SD = 19.9)
Smeulders, 2010	NED	Tele-Interview	TICS	Intervention vs. control: 32.7 (SD = 3.3) vs. 32.4 (SD = 3.1)	–	–	–	–	–	KCCQ	Cardiac Quality of Life	–	–
Vellone, 2015	ITA	Interview	MMSE	23.3 (SD = 6.3)	–	–	–	–	–	Self-Care Heart Failure Index	55.0/100 (SD = 15.7)	53.2/100 (SD = 20.0)	54.0/100 (SD = 20.6)

^a^Scored < 75/100

^b^T-score < 35

^c^Requiring Assistance

^d^Scored > 70/100

^e^Only tested in people with dyspnoea or leg oedema

Country: AUS = Australia, CAN=Canada, ITA = Italy, KOR = South Korea, NED = Netherlands, SWE = Sweden, USA = United States of America

Cognitive testing: 5WIDM = 5 Word Immediate and Delayed Memory test, AFT = Animal Fluency Test, ACE-R = Addenbrooke’s Cognitive Examination, BDT = Block Design Test, BNT = Boston Naming Test, CFT = Complex Figure Test (LDR = Long Delayed Recall), COWA = Controlled Oral Word Association, CVLT = California Verbal Learning Test (SDFR = Short Delay Free Recall, LDFR = Long Delay Free Recall), DSC = Digit Symbol Coding, DSS = Digit Symbol Substitution, FAB=Frontal Assessment Battery, HSCT = Hayling Sentence Completion Test, LNS = Letter Number Sequencing, MOS = Memory Of a Story, PMR = Probed Memory Recall, RAVT = Rey Auditory Verbal Learning Test, RBANS = Repeatable Battery of Assessment of Neuropsychological Status (PN=Picture Naming, SF=Semantic Fluency, FC=Figure Copy, LO = Line Orientation, LL = List Learning, SM = Story Memory, LR = List Recall, LRR = List Recall Recognition, SR = Story Recall, RF = Recall Figure), ROCF = Rey Ostereich Complex Figure, SCWIE = Stroop Colour Word Interference Effect, TMTA = Trail Making Test A, TMTB = Trail Making Test B, Tx = Treatment, VLT = Verbal Learning Test (IR = Immediate Recall, DR = Delayed Recall), VW=Virtual Week, WAIS=Wechsler Adult Intelligence Scale (DS = Digit Span subtest, MR = Matrix Reasoning), WKT = Word Knowledge Test

Assessment of self-care: *EHFScBS* European Heart Failure Self-care Behaviour Scale, *KCCQ* Kansas City Cardiomyopathy Questionnaire

Self-care criteria: *QOL* Quality of life

*NS* Non-significant, *S* Significant

The number of persons with CI varied throughout the studies, ranging from 21 (14%) [31] – 73 (73%) [29]. One study identified 40 (16%) of the study population to have SLUMS test scores consistent with dementia [20].

With regards to testing of individual cognitive domains, two [19, 20] studies tested all five cognitive domains while another three [9, 22, 24] tested four cognitive domains. One [21] study tested three cognitive domains and two [30, 33] tested two cognitive domains. The remaining appraised studies (*n* = 6) assessed global cognition rather than specific cognitive domains.

### Self-care

#### Self-care maintenance

One study [19] examining the influence of cognitive functioning on adherence to treatment recommendations reported non-adherence most commonly arising in diet (32% non-adherent) and exercise (49% non-adherent) recommendations. Less common was non-adherence to clinical appointments (3%) and medication management (1%). In a separate study [22], the same author found people with impaired executive function commonly required assistance with shopping (27%) and food preparation (32%) and less commonly required assistance with transport (8%), medication management (6%) and telephone usage (2%).

Medication adherence in a population of outpatient veterans was impaired in those with MCI when compared to non-CI (70.7% vs. 78.1% *p* = 0.017) subjects [20].

The proportion of patients with adequate self-care maintenance scores on the SCHFI ranged from 50% [29] to 61% [9]. 14% [30] to 36% [9] of patients had adequate self-care management scores and 0% [30] to 44% [9] had adequate self-care confidence scores.

### Impact of cognitive impairment and domains on self-care

Nine studies explored the impact of CI (either global and/or specific domains) on self-care (Table 3).

**Table 3:** Study Outcomes, Impact of Cognitive Impairment, Relevant Risk Factors and Suggested Strategies

Author	Study Outcome (n and/or %)	Impact of Cognitive Impairment on Self- care	Other Risk Factors for Self-Care Impairment	Suggested Strategies/Intervention
Alosco, 2012	Adherence Score: 84.0/100 SD = 11.6. 16% were Non-Adherent^a^	↓Attention:↓Doctor’s Appointment Adherence (r(138) = 0.29, *p* < 0.001) & ↓Medication Management (r(138) = 0.25, *p* < 0.01). ↓Executive Function: ↓Doctor’s Appointment Adherence (r(138) = 0.29, *p* < 0.001). ↓Language:↓Medication Management (r(138) = 0.28, *p* < 0.01) &↓Diet Adherence (r(138) = 0.17, *p* = 0.04)	Myocardial infarction is associated with↑ treatment adherence (ß = 0.23, *p* = 0.01)	Cognitive function assessment can influence the course of heart failure management
Alosco, 2012	Activities of daily living score: 25.2/28 (SD = 3.4)	↓TMTA performance (Attention, Visuospatial): ↓Medication Management (ß = − 0.24, *p* < 0.05) ↓MMSE:↓Driving scores (ß = − 0.25, *p* < 0.001)	–	Regular screening of cognitive impairment can provide information about self-care behaviors
Alosco, 2014	Instrumental activities of daily living score: 13.5/16 (SD = 2.9). ↓Executive function: ↑Cigarette smoking (r(167) = − 0.20, *p* = 0.01)	↓Executive function: ↓Instrumental activities of daily living performance (ß = 0.24, *p* = 0.01) – Especially food preparation (r(167) = 0.16, *p* < 0.03) & medication management (r(167) = 0.15, *p* = 0.05). ↓Executive function associated with ↑cigarette use (r(167) = − 0.20, *p* = 0.01).	Male (ß = − 0.29, *p* < 0.001), Diabetes (ß= − 0.19, *p* = 0.01) Depression (ß = − 0.15, *p* = 0.04) associated with↓instrumental activities of daily living performance	Technological devices which promote executive function could improve self-care outcomes.
Cameron, 2009	Self-care maintenance: 67.8/100, SD = 17.3 Self-care management: 50.1/100, SD = 16.6 Self-care confidence: 62.0/100, SD = 20.0 The 7 variable model^b^ = 39% of variance in Self-care maintenance & 38% of variance in Self-care management	Cognitive function non-significant factor in 7 variable model however when omitted from the model, 6 variables explain ↓4% of the variance in self-care maintenance (39% - > 35%). This was also seen in self-care management (38 - > 34%)	Self-care maintenance: ↑Age: ↑Self-care maintenance (ß = 0.51, *p* < 0.01); Significant comorbidity (CCSI≥4): ↑Self-care maintenance (ß = 0.34, *p* = 0.02). Self-care management: Male: ↓Self-care management (ß = − 0.33, *p* = 0.02); No significant comorbidity (CCSI< 4) (ß = 0.33, *p* = 0.03): ↑Self-care management; Depression: ↑Self-care management (ß = 0.32, *p* = 0.04); ↓Self-care confidence: ↓Self-care management (ß = 0.39, *p* < 0.01)	Screening for modifiable and non-modifiable factors can ↑ health outcomes and follow up strategies
Dickson, 2008	Self-care management: (71.3/100, SD = 18.6) 44% had adequate scores (>70). Self-care maintenance: (71.6/99.99, SD = 14.3) 61% had adequate scores (>70). Significant difference in self-care maintenance and self-care management between expert^c^, novice^d^ and inconsistent groups^e^ (*p* = 0.001).	‘Inconsistent’ group: Cognitive impairment (DSS < 26) had ↑self-care management and ↑self-care maintenance scores vs. ‘↓ vigilant’ and ‘discordant’ (*p* = 0.02 to 0.03).	–	Developing self-efficacy in difficult situations will lead to (+) self-care decisions and help overcome temptations which leads to ↑self-care confidence
Habota, 2015	Trend: Congestive heart failure (mean = 0.5, SD = 0.4) performing ↓ than controls (mean = 0.6, SD = 0.3). For the proportion of tasks missed, there was a main effect of group (F(1,57) = 4.52, *p* = 0.038, ηp^2^ = 0.07). The congestive heart failure group (mean = 0.26, SD = 0.31) missed ↑ tasks than the control group (mean = 0.16, SD = 0.21).	–	–	↑Self-care adherence may need to include prospective memory training
Harkness, 2014	Self-care management: MoCA score < 26 (mild cognitive impairment) scored significantly ↓ vs. scores ≥26 (48.1/100 (SD = 24) vs. 59.3/100 (SD = 22), *p* = 0.035). Also observed with the MoCA cutoff at < 24 and ≥ 24, (45.6/100 (SD = 23) vs. 58.1/100 (SD = 23), *p* = 0.008)	MoCA was a significant factor (B = 1.784, *p* = 0.001) in model for self-care management (F(3,96) = 7.04, *p* < 0.001). Mild cognitively impaired participants (both < 26 and < 24) were ↓ likely to call a doctor or nurse for guidance (52% vs. 89%, *p* = 0.001, 46% vs. 82%, *p* < 0.001 respectively)	–	Formal screening for mild cognitive impairment can help to identify individuals who are risk of self-care management difficulty and of delaying assistance from a health care provider. Experiential learning and problem solving skills are important for the elderly.
Hawkins, 2012	Cognitive impairment present in 57.6%. Verbal learning, immediate memory, and delayed verbal memory were found to be impaired. Associations with cognitive impairment: Age (OR = 1.42, 95%CI = 1.03–1.95, *p* = 0.031); African American race (OR = 3.59, 95%CI = 1.90–6.81, *p* < 0.01); Depression (OR = 1.43, 95%CI = 1.12–1.83, p = 0.004); Former alcohol use (OR = 2.13, 95%CI = 1.06–4.31, *p* = 0.034); missed follow up of pill count (OR = 2.03, 95%CI = 1.20–3.45, *p* = 0.009). Medication adherence ↑ in participants with no CI vs. MCI (78.1% vs. 70.7%, *p* = 0.017)	–	–	Screen patients for cognitive impairment and depression. Interventions should look to target verbal learning, verbal memory and delayed verbal memory
Hjelm, 2015	Psychomotor speed associated with self-care (ß = − 0.09, t(99) = −2.92, *p* = 0.004). No moderating effects of depression were found.	–	–	Screening for impaired psychomotor speed to identify patients in need of individualized self-care teaching.
Karlsson, 2005	Intervention group did not have ↑ knowledge vs. control group after 6 months (13.2 (SD = 3.4) vs. 12.7 (SD = 3.3), NS).	MMSE< 24 had ↓ scores in self-care and heart failure knowledge vs. MMSE≥24 (10.1 (SD = 3.6) vs. 12.8 (SD = 3.4), *p* < 0.01) at baseline. There was no difference between the 2 groups after 6 months.	–	Education of patients should be given individually and given through different means (verbal, written, electronic)
Kim, 2015	NYHA I (asymptomatic) vs. NYHA≥II (symptomatic): Global function (27.8 (SD = 2.5) vs. 24.9 (SD = 4.4), *p* = 0.001), Memory (17.5 (SD = 5.7) vs. 13.4 (SD = 5.2), p = 0.001), executive function (23.4 (SD = 9.8) vs. 16.9 (SD = 9.6), *p* = 0.002) Also observed in self-care confidence (57.0 (SD = 17.4) vs. 53.2 (SD = 13.8), p = 0.009).	Delayed recall memory predicted self-care confidence adequacy (OR = 1.41, 95%CI = 1.03–1.92, *p* = 0.033). MACE had ↓ K-MMSE scores vs. ‘event free’ (23.9 vs. 27.1, t = 2.30, *p* = 0.024).	–	–
Lee, 2013	MoCA < 26: ↓Self-care management scores vs. MoCA ≥26 (difference = 8.2%, SD = 3.8%, *p* = 0.043). MoCA < 24: ↓Adjusted self-care maintenance (difference = 13.8%, SD = 5.4%, *p* = 0.014) and self-care management scores (difference = 21.4%, SD = 8.0%, p = 0.014) vs. participants with scores ≥24. MoCA < 24 also had significantly lower EHFScBS scores (difference = 38.3%, SD = 11.2%, *p* = 0.001)	MoCA < 24 had worse adjusted consulting behavior scores (difference = 50.7%, SD = 15.3%, *p* = 0.001)	–	Cognition should be assessed with clinically appropriate tools (e.g. employing the MoCA cutoff of < 24). Systematic screening for mild cognitive impairment
Smeulders, 2010	Participants with TICS< 33 had worse cardiac quality of life at first follow up (Difference = − 6.3, *p* = 0.027, 95%CI = − 11.9 to − 0.7). Scores were not significantly different at 6 and 12 months.	–	–	Encourage patients with ↓education levels to participate in CDSMP classes. Tailor CDSMP to cognitively impaired patients. Screen for cognitive status and education level.
Vellone, 2015	MMSE score influenced self-care maintenance and self-care management through the mediating effects of self-care confidence MMSE predicted self-care confidence. Self-care confidence predicted self-care management and self-care maintenance. Cognition does not have a direct effect on self-care. It only influenced self-care through its effect on self-care confidence	–	Self-care maintenance ↑Illness duration predicted ↑self-care maintenance Self-care management: ↑NYHA class predicted ↓self-care management Self-care confidence: ↓Age and female gender predicted ↑self-care confidence	Interventions that ↑ self-care confidence may ↑self-care even in patients with cognitive impairment. Reward patients for small successes in their adherence to self-care behaviors. Introduce patients to others in the same situation who are proficient at self-care. Tell patients that they are able to be proficient at self-care. Provide and encourage support for patients.

^a^Scored < 75/100

^b^7 Variable Model constituents: age, gender, comorbidity, cognitive function, depression, social situation, self-confidence

^c^Expert = Proficient at heart failure self-care

^d^Novice = No skill or experience in heart failure self-care

^e^Inconsistent = Neither expert nor novice

CDSMP=Chronic Disease Self-Management Programme, DSS = Digit Symbol Substitution, EHFScBS = European Heart Failure Self-care Behavior Scale, HFK=Heart failure knowledge, HFP=Heart failure program, MACE = Major Adverse Cardiac Event, MMSE = Mini Mental State Exam, MoCA = Montreal Cognitive Assessment, NYHA = New York Heart Association, TICS = Telephone Interview for Cognitive Status, TMTA = Trail Making Test A, (+) = positive, ↑= increased, ↓= reduced

#### Relationship between global cognition and self-care

At the commencement of an educational intervention program for HF patients, patients with an MMSE <24 had lower scores in self-care and HF knowledge when compared to those who had MMSE scores >24 at baseline. However, there was no difference between the two groups after 6 months [16]. Subjects with MoCA scores <24 also had worse consulting behavior scores than their counterparts with scores > 24 [31].

In one study, cognitive function assessed by MMSE score did not significantly predict self-care ability despite contributing to detection of variance in domains of care in the authors’ model [25].

In contrast, Dickson and colleagues [9] demonstrated a significant association of CI (as determined by a DSST score less than <27) [26] with improved self-management and maintenance scores. Further, MoCA scores were significant for predicting self-care management abilities with subjects scoring < 26 being less likely to call a doctor or nurse for disease management guidance [29]. Potentially impacting self-care, subjects with a history of major adverse cardiac events had lower K-MMSE scores compared to those who were event free [30].

A summary of the influence of specific cognitive domains on self-care is presented in Table 3.

### Other risk factors for self-care impairment

Other factors related to impairment in self-care were investigated in five studies (*n* = 5) (Table 3).

History of myocardial infarction was found to be protective for overall adherence to medication [19]. Additionally, male gender and having a comorbid diagnosis of depression or diabetes was predictive of lower IADL scores [22]. Furthermore, severe (NYHA) grades of HF were associated with reduced self-care management.

Cameron et al. [25] identified potential factors associated with each of the three domains of self-care. Better self-care maintenance was predicted by greater age and presence of a moderate to severe comorbidity. Improved self-care management was associated with presence of a significant comorbidity and high self-care confidence Finally, poor self-care management was related to male gender. This study only included 50 subjects of which *n* = 18 had a MMSE<27 so results should be interpreted with caution.

## Discussion

The impact of CI in patients with HF is significant, contributing to poor engagement in self-care leading to worse health outcomes and increased mortality. By elucidating the relationship between impairment in specific cognitive domains, self-care as well as identifying factors that may modulate self-care abilities, clinicians may tailor management accordingly. Barring patients with CI from participating in their own management is simplistic, disrespectful and may be counterproductive, increasing dependence and caregiver stress [14, 34, 35].

### Statement of key findings

Poor cognition in patients with HF is well recognized and considered to be a result of chronic cerebral hypo-perfusion, leading to ischemic damage and subsequent functional alteration [10]. Optimal self-care is an important non-pharmacological aspect of HF management that stabilizes symptoms and improves health outcomes.

To our knowledge, this is the only systematic review to consider the role of CI, from the spectrum of mild CI to dementia, on self-care in community dwelling adults with HF. Throughout the appraised articles there was heterogeneity in the methods used to assess cognition and self-care. As a consequence, the results of appraised studies could not be analysed in an aggregate form.

### Self-care domain adequacy in cognitive impairment

When assessment was based on the SCHFI self-care assessment tool, self-care management and self-care confidence adequacy was lacking in CI subjects with self-reported adequacy.

Interestingly, Vellone and colleagues suggest self-care confidence is impaired by poor cognition thus leading to worse self-care behaviours [32]. Dickson and colleagues also found that self-efficacy and positive attitudes towards disease were important in facilitating appropriate or “*expert”* self-care behaviours [9].

Of note, the proportion of participants with adequate self-care maintenance scores were equal, if not higher in CI subjects compared to those who had inconsistent levels of cognition [9, 29]. MCI subjects had lower medication adherence rates than subjects with no CI, but similar rates to those with increasingly worse CI [20]. This may be attributed to CI persons having strong social support networks and assistance, which has been shown to predict greater adherence to treatment in populations with cardiac disease [36]. Unfortunately, none of the studies appraised analysed the effect of caregivers or spouses on adherence in the population of interest.

### Cognitive impairment and lifestyle adherence

Patients who either had impairments in multiple separate domains or global cognition had poor self-care maintenance abilities. These were namely medication adherence, compliance with lifestyle recommendations or requiring assistance with ADLs [19–22]. The impact of cognition on these aspects of self-care is important as it determines the execution of these key activities. For instance, medication management and driving are inextricably linked to outcomes such as re-hospitalisation or admissions to geriatric units respectively [22].

One proposed theory for impaired self-care ability is that as cognitive decline diminishes so does functional ability with the resulting lessened influence of personal values towards self-care [9, 37]. Specifically, cognitive domains implicated included attention/information processing, executive function, language and finally, visuospatial and constructional abilities. Attentional control and executive functioning are domains often impaired in most chronic, systemic diseases [38–40].

Given the published literature [41] it was expected that impaired executive function is linked to inability to self-care. Executive functioning is important as it is related to dis-inhibition, poor self-monitoring, poor organisation and planning and also affects learning and recall efficiency. Impairment in this domain affects the critical need for HF patients to be able to adapt to complex treatment and lifestyle regimens, to recognise and respond to worsening symptoms (e.g. fluid overload, shortness of breath), communicate and seek help in a timely manner, have insight into disease (hence higher rates non-adherence to cigarette smoking) and ability to conduct multiple daily self-management tasks [42, 43]. Therefore, deficits in executive function are known to be associated with a lack of both awareness about worsening symptoms and timely decisions ultimately leading to poorer outcomes, including decompensation and hospitalization [44].

Decline in language function is related to poor literacy, inability to state concerns about disease condition and, poor understanding of instructions and medical advice. All of these, along with executive function decrements may also contribute to worse treatment and lifestyle adherence in those with CI and HF.

If attention and poor concentration are an issue [9] it may distract from execution of certain tasks while impairment in prospective memory may have an adverse impact on engagement in self-care behaviours such as picking up prescriptions from the pharmacist, attending clinical appointments, treatment adherence and daily weighing, all of which are important in HF self-management [33].

Impairments in psychomotor speed may result in poor flexibility in shifting activities and slowing of responses to visual stimuli. These skills are important in learning and conducting multiple daily self-care tasks [24, 43].

Consistent with the relationship between CI and self-care with poor outcomes demonstrated by the majority of appraised studies, Pressler and colleagues reported that along with reduced LVEF (≤40%), impairment in global CI, memory, psychomotor speed and executive function were predictors of 12 month all-cause mortality [45].

Symptoms of HF are difficult to interpret even in cognitively intact individuals. This is increasingly difficult in the context of impaired cognitive domains and is compounded by the pathophysiology of HF decompensation where symptoms of fatigue or acute confusion may detract from executing effective self-care actions [46]. Reduced ability to self-care will subsequently lead to worsening symptoms and advanced cardiac dysfunction.

### Seeking help

Subjects with poor MoCA scores were less likely to seek assistance from a medical staff for disease management guidance respectively [29, 31]. Executive function deficits may impair recognition of symptoms and problem-solving, hence these patients delay initiation of self-management and may not recognize when, why or from whom they need to seek assistance. This is further complicated by IADL, language and attention deficits as HF patients may not have the ability to engage in using communication facilities [29].

### The effect of depression on self-care

Psychological status influences self-care behaviors [47] through patient perceived self-efficacy or indirectly, through effects on memory and executive function [48]. In the present review, a diagnosis of depression was found to be predictive of lower IADL abilities and poor self-care management [22, 25].

### Education programs

One study explored the effectiveness of an education program [16]. CI patients had lower scores in self-care and HF knowledge initially compared to non-CI subjects. However, there was no difference in self-care and knowledge after 6 months of the program. This may be due to improvement in cardiac function and hence cognitive function in patients who were receiving acute treatment for HF [49]. However, several studies have also identified that provision of education, treatment and lifestyle instructions alone are not adequate to uphold appropriate self-care behavior [50, 51].

### Strengths and limitations

The current review is extensive, examining the effect of CI on a spectrum of mild-severe, covering literature published from 2000 to March − 2016. We were limited to peer reviewed literature published solely in the English language. Ten of the 14 studies appraised were cross-sectional studies, however, prospective studies may more accurately explore the causal nature between CI and self-care among patients with HF.

A stronger relationship between cognition and self-care may not have been observed due to the use of certain cognitive testing tools which are insensitive to higher order functions. If clinicians choose to screen for dementia with the MMSE, they may possibly fail to detect mild impairments in higher functioning. In the clinical setting and indeed for future research this issue may be circumvented by utilizing ‘executively focused’ neuropsychological batteries in addition to more commonly used screening test tools [52]. Future studies may consider a meta-analysis design to gain power to further elucidate a relationship between CI and self-care.

A major limitation of the studies reviewed is that assessment of adherence to lifestyle recommendations and answers to the SCHFI were self-reported. Okonkwo and colleagues [53] identified that patients with memory impairments, a domain commonly impaired in HF, tend to over estimate their abilities in completing daily living tasks, which is relevant for two studies which reported adherence to treatment regimens or lifestyle recommendations [19, 21].

Studies with inclusion criteria of EF < 45% are better in terms of selecting moderate to severe systolic dysfunction. The others that had a wide range of ejection fraction (including mild and low normal ejection fraction) could have a diluted effect of severity – as it would not be expected that patients with an ejection fraction of 50% (low normal) would have similar self-management issues or similar re-admission rates for decompensations as those with ejection fraction 30%.

A lack of studies exploring the impairment of specific cognitive domains or dementia subtypes (e.g. vascular, frontotemporal etc) and their involvement in all aspects of self-management makes it difficult to definitively identify the most effective recommendations to manage CI persons with HF.

### Implications for health policy

Persons with CI and HF require more resources and support in the community to carry out self-care tasks compared to their non-CI counterparts. Primary care and community services should be re-designed to evaluate and cater to individual’s self-care needs. The relationship between CI and self-care ability in HF is quite prominent, however, effectiveness of programs to assist those with CI and their carers needs to be further elucidated. Programs may have differential benefits based on cognition, support and demographic factors so these need to be further characterized to improve management and outcomes for these persons in the community. Table 4 outlines advice generated for clinical use.

**Table 4:** Advice for Clinical Management of Patients with Heart Failure and Cognitive Impairment

Task	Sub Task	Impairments	Recommendations
Understanding and Monitoring symptoms	Education Programs	Patients with better cognitive function may benefit more from self-management programs than those with worse cognition in the short term [17]. Those with lower educational status may benefit more from programs. Poorly educated subjects may be less skilled with respect to self-management at baseline and hence may have more to learn from such programs [54–56].	Clinicians should consider baseline education status to deliver information appropriately as well as ascertain the benefit patients with HF and CI may obtain by undertaking self-management programs. However, several studies have also identified that provision of education, treatment and lifestyle instructions alone are not adequate to uphold appropriate self-care behavior [50, 51],
	Seeking Help	Poor global cognition correlated with worse consulting behaviors [29, 31]. Making decisions to seek help is complex and requires an understanding of HF. Executive function deficits in CI subjects may impair recognition of symptoms and problem-solving hence may delay initiation of self-management as well an inability to recognize who, when or why they need to seek assistance. HF patients with deficits in IADL, language and attention deficits may not have an ability to engage in communication facilities (e.g. telecommunications, driving to the clinic, making appointments online or by phone) [29].	Clinicians should be aware of the impact of executive function on communication difficulties for persons with HF and CI. Cognitive tests geared towards executive function assessment should be utilized. Clinicians should provide resources for and communication solutions for allow easy access to healthcare for persons with HF and CI Teaching patients select few response options for clinical scenarios may provide a baseline to refer to when a response is required spontaneously Provision of in-home prompts including wall calendars, blister packs, management flow charts etc. Where possible provide home visits or an escort to clinical appointments Establishing an appointment and healthcare support routine that does not vary.
Adherence to Lifestyle and Treatment	Psychological Status	Psychological status has been demonstrated to have an influence on self-care behaviors [47] through patient perceived self-efficacy or indirectly, through effects on memory and executive function [48]. A diagnosis of depression was found to be predictive of lower IADL abilities and self-care management [22, 25].	Clinicians may benefit from screening for and appropriately treating depression in patients with heart failure in order to prevent the associated adverse affects it may have on self-care.
	Personal motivation	Cognitive decline not only diminishes functional abilities, it may dampen the influence of personal factors related to self-care [9, 37]. These include belief in treatment of the disease, information sources, personal and cultural values that would otherwise influence self-care in a positive manner.	Clinicians should endeavor to convey how health care goals may serve the patient’s personally valued goals and priorities in life.
	Cognition	Patients who either had impairments in multiple separate domains or global cognition had poor self-care maintenance abilities. These were namely medication adherence, compliance with lifestyle recommendations or requiring assistance with ADLs.	By elucidating the relationship between impairment in specific cognitive domains and self-care as well as identifying factors that may modulate self-care abilities, clinicians may tailor management.
Managing Other Medical Conditions		Having a comorbid disease was related to better management and maintenance behaviours [25]. Patients being well versed with and used to self-care practices or, where increasing symptoms or reduced functional capacity may motivate self-care behaviours. Increased burden of comorbidities and symptoms may be detrimental for patients. Increased symptoms burden may limit functional capacity and that could lead to increasing social support.	Clinicians should be aware of pre-existing disease which may aid patients who are well versed in self-management or in contrast, may detract from management of concurrent illness or where symptom burden may hinder self-care abilities. Multidisciplinary and multispecialty input may be required to ensure appropriate management of comorbid conditions.
General Self-Care Behaviors		Self-care confidence that was impaired by poor cognition thus leading to worse self-care behaviours [32]. Self-efficacy and a positive attitude towards disease was important in facilitating appropriate or “*expert”* self-care behaviours [9].	Clinicians may target confidence through problem solving and experiential learning in HF patients with CI may improve self-care functions even in the context of cognitive decline [57].

### Generalizability

The aforementioned findings may be applied widely at the patient level as demographic characteristics of subjects were largely similar where impairments in cognitive domains were not based on geography or ethnicity. The present review includes articles spanning twelve years (2005–2016), therefore assessment and interpretations of CI, as well as the diagnostic criteria for dementia/CI may have varied across time.

## Conclusion

Managing persons with HF and CI is particularly difficult. Decrements in cognitive domains adversely impacts self care abilities of these individuals, ultimately leading to poor outcomes. Clinicians need to be aware of the differential impacts of impairments in cognitive domains and tailor their management accordingly. Regular screening tests for higher order functions along with those for global cognitive function in the older patients with HF are necessary if optimum self-care is to be supported. Awareness of other factors such as depression, self-confidence and access to supports may also modulate self-care ability. A holistic, multifactorial approach is required to improve outcomes in this particularly vulnerable population with HF and CI.

## Additional file

Additional file 1: **Table S1.** DSM V Criteria for Diagnosing Major & Minor Neurocognitive Disorder (NCD)*. **Table S2.** Search terms used for literature search. **Table S3.** Conversion of scales reporting severity of comorbid conditions [58–60]. **Figure S1.** PRISMA flow diagram of identification, screening, and inclusion of eligible articles. (DOCX 49 kb)

## Abbreviations

ADLs: Activities of daily living
CI: Cognitive impairment
DSST: Digit symbol substitution test
EHFScB-9: European heart failure self-care behaviour scale
HF: Heart failure
IADLs: Independent activities of daily living
KCCQ: Kansas City cardiomyopathy questionnaire
K-MMSE: Korean version mini mental state examination
LVEF: Left ventricular ejection fraction
MMSE: Mini mental state examination
MoCA: Montreal cognitive assessment
NYHA: New York heart association
SCHFI: Self Care of Heart Failure Index
SLUMS: St. Louis University mental status exam
