# Supplementary material for: Self-management of heart failure in dementia and cognitive impairment: a systematic review
Source: BMC Cardiovasc Disord. 2019 Apr 29;19:99. doi: 10.1186/s12872-019-1077-4 (PMC6489234; doi:10.1186/s12872-019-1077-4)
Supplement: Supplementary file 1 — Table S1. DSM V Criteria for Diagnosing Major & Minor Neurocognitive Disorder (NCD)*. Table S2. Search terms used for literature search. Table S3. Conversion of scales reporting severity of comorbid conditions [58–60]. Figure S1. PRISMA flow diagram of identification, screening, and inclusion of eligible articles. (DOCX 49 kb) [file 12872_2019_1077_MOESM1_ESM.docx]

**Additional file 1**

**Self-management of Heart Failure in Dementia and Cognitive Impairment: A Systematic Review**

Janaka Lovell^1^, Tony Pham^2^, Samer Q Noaman^3^, Marie-Claire Davis^4^, Marilyn Johnson^5^ Joseph E Ibrahim^6^

^1^ MD, BBiomedSc. Department of Forensic Medicine, Monash University, Southbank, Victoria 3006, Australia

^2^ BBiomedSc, Department of Forensic Medicine, Monash University, Southbank, Victoria 3006, Australia

^3^ M.B.Ch.B., Department of Cardiology, Alfred Health, Victoria 3004, Australia.

^4^ BPsych (Hons), MPsych (ClinNeuro), Calvary Health Care Bethlehem, Victoria 3162, Australia

^5^ BA (Hons), MAppSocRes, PhD. Institute of Transport Studies, Monash University, Victoria 3800, Australia

^6^ MBBS, GradCert HE, PhD. Department of Forensic Medicine, Monash University, Southbank, Victoria 3006, Australia

**Corresponding Author**

Dr Janaka Lovell

Department of Forensic Medicine, Monash University, 65 Kavanagh Street, Southbank, Victoria 3006, Australia.

Telephone number: +61 448889438

Email address: [janakalovell@gmail.com](mailto:janakalovell@gmail.com)

**APPENDIX**

**Table S1. DSM V Criteria for Diagnosing Major & Minor Neurocognitive Disorder (NCD)***

| **Cognitive Domain**† | **Description** |
| --- | --- |
| Complex attention | Includes sustained attention, divided attention, selective attention and information processing speed. |
| Executive function | Includes planning, decision making, and working memory, responding to feedback, inhibition and mental flexibility. |
| Learning and memory | Includes free recall, cued recall, recognition memory, semantic and autobiographical long term memory, and implicit learning. |
| Language | Includes object naming, word finding, fluency, grammar and syntax, and receptive language. |
| Perceptual-motor function | Includes visual perception, visuoconstructional reasoning and perceptual-motor coordination |
| Social cognition | Includes recognition of emotions, theory of mind and insight. |

* Dementia newly defined as Major NCD; CI newly defined as Minor NCD in DSM-V

† Cognitive domains retrieved from *https://fightdementia.org.au/files/helpsheets/Helpsheet-DementiaQandA11-DiagnosticCriteriaForDementia_english.pdf*

**Table S2. Search terms used for literature search**

| **Concept 1: Dementia** | **Concept 2: Heart Failure** | **Concept 3: Aged** | **Concept 4: Self-management** |
| --- | --- | --- | --- |
| Cognition disorder | Heart Failure | Aged | Person |
| Cognitive | Heart Disease | Middle aged | Self |
| Neurocognitive disorder | Cardiovascular Disease |  | Care |
| Cognitive defect | Coronary Artery Disease |  | Secondary prevention |
| Cognitive impairment |  |  | Manage |
| Confusion |  |  | Disease management |
| Dementia |  |  |  |
| Memory disorder |  |  |  |
| Alzheimer disease |  |  |  |
| Parkinsonian disorders |  |  |  |
| Parkinson |  |  |  |

**Table S3. Conversion of scales reporting severity of comorbid conditions**

| **Converted scale for severity of comorbidities** | **Corresponding reported scales and scores for severity of comorbidities** |
| --- | --- |
| Mild | - Charlson comorbidity index (58) : 1–2. - Chess score (59): 0-1. - Chronic comorbidity count (60) : 0-1. - Number of comorbidities/15: 0-5. |
| Moderate | - Charlson comorbidity index: 3–4. - Chess score: 2-3 - Chronic comorbidity count: 2-3. - Number of comorbidities/15: 6-10. |
| Severe | - Charlson comorbidity index: ≥5. - Chess score: 4-5 - Chronic comorbidity count: ≥4. - Number of comorbidities/15: 11-15. |

**Figure S1. PRISMA flow diagram of identification, screening, and inclusion of eligible articles**

Records identified through database search

**n= 21688**

Number of full-text articles assessed against eligibility criteria:

1.Original research in peer-reviewed journal

2.Published 1 Jan 2000 – 3 Mar 2016

3.Studies in English language

4. Population being community dwelling elderly

5.Examination of the impact of dementia or cognitive impairment and association of specific cognitive domains on self-management in elderly heart failure subjects

**n=46**

Number of records excluded from title and abstract screening

**n=10642**

Number of records screened

**n=10688**

Date Limit 2000 – 16^th^ March 2016

**n=1814**

Number of full-text articles excluded

Abstract only= 4

Cognitive impairment and self-care not examined= 2

Elderly excluded= 1

Looked at heart failure profile=1

Heart failure and dementia not primary exposure= 11

Measuring effect of other chronic disease +/- HF= 1

No comparison group= 2

Not in peer reviewed journal= 3

Testing cognitive measurement tool= 1

Full text not available= 2

Not community dwelling = 4

**Total n= 32**

Original research studies examining self-care and cognitive domains impaired in heart failure patients with a co-morbid dementia syndrome

**n= 14**

Number of duplicate records removed

**n=9186**
